# Supplementary material for: Harnessing digital technology to improve agricultural productivity?
Source: PLoS One. 2021 Jun 28;16(6):e0253377. doi: 10.1371/journal.pone.0253377 (PMC8238233; doi:10.1371/journal.pone.0253377)
Supplement: S1 Fig — (DOCX) [file pone.0253377.s001.docx]

Sowing Period

Standing Crop

Harvesting Period period

**Baseline Survey 2013**

**Crop cycle 1**

June-July 2013

June-July-October 2012

January-March 2013

May-June 2013

**Experimental**

**intervention**

Random assignment of KCC hotline number to farmers

June-October 2013

**Endline Survey 2014**

June-July 2014

Sowing Period

**Crop cycle 2**

June-July-October 2013

Standing Crop

January-March 2014

May-June 2014

Harvesting Period period

S1 Fig. Timeline of the intervention
