# Supplementary material for: Harnessing digital technology to improve agricultural productivity?
Source: PLoS One. 2021 Jun 28;16(6):e0253377. doi: 10.1371/journal.pone.0253377 (PMC8238233; doi:10.1371/journal.pone.0253377)
Supplement: S1 Table — (DOCX) [file pone.0253377.s002.docx]

S1 Table. Selected variable definition in regression analysis

| Variable name | Definition |
| --- | --- |
| Treated (A) | Dummy =1 for households that were randomly assigned to the group intended to be treated in the field experiment, and 0 otherwise. |
| Time (T) | Dummy =1 for the year 2014, and value 0 for the year 2013. |
| Education | Category based on number of years of education. |
| Crop experience | Category based on number of years of crop experience. |
| Caste | Dummy = 1 for a household that belongs to scheduled caste and tribe, otherwise 0. |
| Land owned | Log of farmland owned in acres. |
| Crop yield | Crop production divided by area cultivated. |
| Pigeon pea | Dummy = 1 for pigeon pea crop grown, otherwise 0. |
| Finger millet | Dummy = 1 for finger millet crop grown, otherwise 0. |
| Horsegram | Dummy = 1 for horsegram crop grown, otherwise 0. |
| Paddy | Dummy = 1 for paddy crop grown, otherwise 0. |
| External agricultural information | Dummy=1 for agricultural information from public and private sources, other than the helpline sources, otherwise 0. |
| Public ext. advisor visit | Category based on some visits of the Public Extension Advisor. |
| Distance to GP | Category based on road distance from farmer’s house to the local Government administrative division (GP). |
| Distance to *Taluk* | Category based on road distance from farmer’s house to the *Taluk* (sub-district town). |
| Total asset value | Log of the value of the durable assets owned by the household in Indian rupees (house, television, radio/transistor, mobile phone/telephone, steel trunk/almirah, car/van/jeep, motorbike/scooter, bicycle, VCD). |

Notes: Public Sources include *Rayata Samparka Kendra* (RSK- Agricultural Information Centre) and *Krishi Vignana Kendra* (KVK – Agricultural Science Centre). These are Local Government Centres providing agricultural information. Private Sources include NGO, Agricultural college, Co-operative society, Media (i.e. Radio/ T.V./ Newspaper), and Farm magazines like *Annadata*, *Krishimunnade*, *Krishimitra* or *Sirisamrudhhi*. The data for crop yields are generated from primary farm surveys.
