# Supplementary material for: Harnessing digital technology to improve agricultural productivity?
Source: PLoS One. 2021 Jun 28;16(6):e0253377. doi: 10.1371/journal.pone.0253377 (PMC8238233; doi:10.1371/journal.pone.0253377)
Supplement: S2 Table — (DOCX) [file pone.0253377.s003.docx]

S2 Table. Differential effect of shocks in the second crop cycle across treatment groups

| Variable | Control Mean | Treatment Mean | P-value |
| --- | --- | --- | --- |
|  | (1) | (2) | (3) |
| Weather shock (1 –normal rainfall; 0 - otherwise) | 0.98  (0.01) | 0.96  (0.01) | 0.38 |
| Pest / disease shock (1 – yes; 0 – no) | 0.59  (0.05) | 0.48  (0.03) | 0.11 |
| Weed infestation (1 – yes; 0 – no) | 0.12  (0.03) | 0.20  (0.03) | 0.12 |

Notes: Weather shock refers to the rainfall shock to output based on the response to the question answered by the farmers – how was the rainfall in the last agricultural year? Pest/disease shock is based on the farmer’s response to the question – did the crops suffer loss from pest attack or any disease in the last agricultural year? Weed infestation is based on the response from farmers on the question – did the crops suffer from weed infestation in the previous agricultural year?
