# Supplementary material for: Harnessing digital technology to improve agricultural productivity?
Source: PLoS One. 2021 Jun 28;16(6):e0253377. doi: 10.1371/journal.pone.0253377 (PMC8238233; doi:10.1371/journal.pone.0253377)
Supplement: S3 Table — (DOCX) [file pone.0253377.s004.docx]

S3 Table. Hotline usage

| Information type | Usage of information | | | | | |
| --- | --- | --- | --- | --- | --- | --- |
|  | Mean calls  (number) | | | Effectiveness of response (percentage) | |  |
|  | (1) | | | (2) | |  |
| Weather forecast | 15 | | | 60 | |  |
| Input use – seed variety | 20 | | | 40 | |  |
| Input use – fertiliser | 23 | | | 63 | |  |
|  | Pigeon pea |  | Non-pigeon pea |  | |  |
|  | (3) |  | (4) |  | |  |
| Input use – sprays | 25 |  | 21 | 80 |  |  |
| Cultivating practices | 45 |  | 12 | 90 |  |  |
| Pest and diseases | 42 |  | 18 | 92 |  |  |

Notes: Cultivating practices excludes pest and diseases which are shown separately. Mean calls refer to the number of calls all treatment farmers made in aggregate to seek information for each of the information types. The effectiveness of the response shows the self-reported percentage of farmers who responded ‘yes’ under each of the information types. The responses were recorded as ‘yes’ (code 1) and ‘no’ (code 0) on the question: was the advice received useful for each of the information you pursued?
