# Supplementary material for: Harnessing digital technology to improve agricultural productivity?
Source: PLoS One. 2021 Jun 28;16(6):e0253377. doi: 10.1371/journal.pone.0253377 (PMC8238233; doi:10.1371/journal.pone.0253377)
Supplement: S4 Table — (DOCX) [file pone.0253377.s005.docx]

S4 Table. Control and treatment households – baseline balance check -2013

| Variable | Control mean | Treatment mean | P-value |
| --- | --- | --- | --- |
| **Panel A: Farmer characteristics** | | | |
| Family size (number of members) | 5.1  (2.44) | 4.76  (1.96) | 0.51 |
| Age in number of years | 49.1  (11.10) | 50.60  (12.36) | 0.55 |
| Education (number of years of education) | 5.70  (4.74) | 6.28  (4.55) | 0.57 |
| Caste (1– belonging to scheduled caste; 0 – otherwise) | 0.13 (0.35 ) | 0.21 (0.41) | 0.37 |
| Crop experience (number of years in crop farming) | 30.1  (10.49) | 31.60  (12.32) | 0.54 |
| Land owned in acres | 5.13  (3.16) | 5.45  (3.88) | 0.67 |
| Total land cultivated in acres | 5.30  (3.04) | 5.52  (3.88) | 0.75 |
| Total land irrigated in acres | 1.95  (2.15) | 1.86  (2.60) | 0.86 |
| **Panel B: Farm plot characteristics** | | | |
| Crop yield per acre | 2.58  (4.31) | 1.82  (4.59) | 0.27 |
| Pigeon pea yield per acre | 0.722  (1.54) | 0.255  (0.56) | 0.25 |
| Finger millet yield per acre | 2.36  (3.15) | 1.58  (2.47) | 0.07 |
| Land under pigeon pea in acre | 0.75  (0.57) | 0.59  (0.44) | 0.31 |
| Land under finger millet in acre | 1.43  (0.83) | 1.59  (1.09) | 0.43 |
| Cultivation cost per acre– plowing (Indian Rupees) | 1166.48  (169.50) | 1114.78  (94.76) | 0.79 |
| Cultivation cost per acre – harrowing (Indian Rupees) | 124.25  (27.31) | 140.55  (21.16) | 0.63 |
| Cultivation cost per acre – sowing (Indian Rupees) | 774.80  (74.55) | 981.02  (114.53) | 0.13 |
| Cultivation cost per acre – interculture (Indian Rupees) | 341.56  (64.78) | 245.84  (25.06) | 0.17 |
| Cultivation cost per acre – fertilizer (Indian Rupees) | 2255.56  (186.79 | 2335.58  (187.43) | 0.76 |
| Cultivation cost per acre – manure (Indian Rupees) | 829.84  (393.88) | 1323.40  (342.96) | 0.34 |
| Cultivation cost per acre – insecticide (Indian Rupees) | 105.35  (23.62) | 185.13  (51.59) | 0.16 |
| Cultivation cost per acre – total (Indian Rupees) | 11721.22  (1102.13) | 10861  (915.50) | 0.54 |
| Revenue per acre (Indian Rupees) | 11542.51  (3348.99) | 6126.33  (1148.90) | 0.13 |
| Weather shock (1 –normal rainfall; 0 - otherwise) | 0.94  (0.02) | 0.97  (0.01) | 0.37 |
| Pest / disease shock (1 – yes; 0 – no) | 0.25  (0.05) | 0.18  (0.03) | 0.31 |
| Weed infestation (1 – yes; 0 – no) | 0.27  (0.05) | 0.18  (0.03) | 0.21 |
| **Panel C: Source of farm information** | | | |
| Number of visits of the public extension advisor per season | 1.16  (0.53) | 1.20  (0.56) | 0.74 |
| Source of crop information: public/private | 0.26  (0.45) | 0.25  (0.44) | 0.86 |
| Household road distance to GP in km | 4.74  (2.61) | 4.07  (2.32) | 0.23 |
| Household road distance to state / national highway in km | 6.76  (3.29) | 5.69  (3.19) | 0.14 |
| Household road distance to sub-district town in km | 21.4  (8.21) | 21.95  (7.85) | 0.76 |
| **Panel D: Household wealth** | | | |
| Log(Total asset value)(i.e. household durables value) | 10.79  (1.63) | 11.03  (2.28) | 0.56 |
| House owned | 0.53  (0.51) | 0.60  (0.49) | 0.53 |
| Car owned | 0.03  (0.18) | 0.03  (0.17) | 0.92 |
| Bike owned | 0.56  (0.56) | 0.60  (0.55) | 0.76 |
| Television owned | 0.76  (0.43) | 0.76  (0.42) | 0.98 |
| Radio owned | 0.06  (0.25) | 0.014  (0.12) | 0.29 |
| Bi-cycle owned | 0.96  (0.66) | 0.83  (0.66) | 0.38 |
| Telephone/ mobile owned (number) | 1.26  (0.98) | 1.17  (0.62) | 0.64 |

Notes: H0: mean (Treatment) – mean (Control) = 0. Wild bootstrap P-values are calculated using clustering at GP level. When P-value is not significant, we do not reject H0. It means the treatment and control group on an average is similar to each other in tested attributes. Land cultivated is greater than land owned due to the prevalence of sharecropping practices. Also, some area under field crops is mixed with plantation crops such as Coconut, Banana, and Arecanut. Therefore, the area under crop cultivation could be less than owned / overall cultivated land.
