# Supplementary material for: Harnessing digital technology to improve agricultural productivity?
Source: PLoS One. 2021 Jun 28;16(6):e0253377. doi: 10.1371/journal.pone.0253377 (PMC8238233; doi:10.1371/journal.pone.0253377)
Supplement: S5 Table — (DOCX) [file pone.0253377.s006.docx]

S5 Table. Summary of the information received from the KCC helpline

| S. No | Problem/Issue | Reason | Advice received to deal with the problem |
| --- | --- | --- | --- |
| 1 | Land Preparation: finger millet and pigeon pea cultivated as rainfed crops. Farmers do not adopt soil and moisture conservation practices to achieve stability in yield. | Farmers generally do shallow ploughing and do not harrow the field 2-3 times. | Explained the farmers to plough the field deeply to get fine tilth for moisture conservation. |
| 2 | Farmers often save the seeds of the previous season (homegrown) and use them year after year for planting. | Farmers are unaware that repeated cultivation of same seeds cause yield decline and are sensitive to pests and diseases. | Advised farmers to select/change seeds/varieties for higher yields. Recommended to improved varieties like L5, HR911, and MR1, and directed farmers to get the seeds from RSK or any government agencies. |
| 3 | Most of the farmers grow finger millet long duration varieties, and they are not suitable if rainfall is delayed. | Farmers have no idea about improved/high yielding short duration varieties that can meet the specific requirements of their region. | Explained to the farmers that in case of delayed rainfall, short-duration variety is the best to realise some yields. Some of the short duration varieties are GPU-45, GPU-48, GPU-26, Indof-9, and KMR-204. They were also suggested to go for Mustard or horsegram if there is a delay in monsoon. |
| 4 | Seed Treatment: Farmers generally do not follow seed dressing with fungicides/biocontrol agents. | Farmers use only homegrown seeds. As the season starts they plant them without treating the seeds with chemicals. They also don’t realise that treated seeds are resistant to fungal diseases during later stages. | Motivated most of the farmers to adopt seed treatment. Seed treatment under assured rainfall/water condition provides initial strength and vigour to the crops. Treat the seeds with Carbendazim or Thiram at 2g/kg of seed 24 hrs before sowing. Treated seeds will control the incidence of finger millet neck/finger blast and fusarium wilt in pigeon pea. Seed treatment with Trichoderma *viridi* for pigeon pea (8 gm/kg of seed). |
| 5 | Organic Fertilizer:  Improper usage of farmyard manure (FYM) in plantation and field crop fields. | Farmers throw FYM in their fields and leave them uncovered without properly incorporate into the soil. | Explained farmers about the proper application of FYM. Spread FYM evenly on the unploughed field and then plough and incorporate in the soil. If the manure is left uncovered in the field, then all the nutrients will be lost. |
| 6 | Decreased yield due to moisture stress at the time of flowering and fruiting (finger millet and pigeon pea). | Farmers are not aware of any foliar sprays which mitigate moisture stress. If inadequate rainfall and water shortage, the crops start drying and produce no yield. Farmer’s leave the field for grazing. | Explained to the farmers about this mid-season management practice. The recommendation is the foliar spray of 2% KCL (2ml in 100 water) during dry spells. |
| 7 | Farmers do not follow the timely application of Nitrogen in finger millet. Based on the soil test report, soils are deficient in Nitrogen and micronutrients. Nitrogen is the major input to increase crop output. | Not aware of the proper use of fertiliser and micronutrients. | Soil analysis based nutrient recommendations were provided at GP-wise for selected crops. Urea one bag at vegetative stage (25 days after planting) and before flowering. Advised farmers that under assured rainfall conditions, supplementation with micronutrients such as ZnSo4, Boron, and Gypsum along with a recommended dose of fertiliser will undoubtedly increase crop yields. |
| 8 | Weed infestation at the time of vegetative and flowering stages in finger millet, pigeon pea, and paddy. | Most farmers do one time weeding at the early stages of crop growth. | Explained to the farmers that if weeds are not controlled, then yields will decrease by 30%. Cultural and mechanical measures can effectively manage weed infestation. Inline sown crop, 2-3 inter-cultivations and hand weeding are required. In broadcast crop, two effective hand weeding will minimise weeds. Farmers are also told not to allow weeds to produce flowers. If flowering sets in, seeds will fall to the ground and continue to pop up year after year. |
| 9 | Intercropping/Mixed cropping. No change in intercropping pattern/crop combinations. | Farmers follow traditional way of intercropping pattern, i.e., finger millet and field bean, and use the produce for home consumption. | Suggested farmers to adhere to profitable/remunerative crop combinations. For example, finger millet and maize (8:2), finger millet and black/green gram (8:2). These combinations will minimise the occurrence of disease and pest. Finger millet and mustard are ideal crop combinations. Mustard flowers bloom during early stages of finger millet crop and attract ladybird beetle, a predator of aphids attacking finger millet crop. In case of rain failure, mustard crop acts as an insurance crop. |
| 10 | The incidence of pests in finger millet and pigeon pea. Mite problem is severe in finger millet when the crop suffers from drought at early stages. Whereas, pod borer is a significant pest in pigeon pea affecting yield drastically. | Farmers are wary of using chemicals/pesticides as the crops are mainly grown for their home consumption. | Explained the use of cost-effective and eco-friendly ways to control these pests. Soap water spray is an ideal way to control mites in finger millet. Helicoverpa larvae (pod borer) can be manually removed by shaking the plants and collecting the larvae and destroying them. Neem oil spray (1 to 2 ml per Litre) or Profenophous (2ml/L) are effective in controlling pod borer in pigeon pea. |
| 11 | Prevalence of Fusarium wilt and sterility mosaic disease leading to yield decline in pigeon pea. | Lack of awareness among farmers with regards to wilt. The disease appears as gradual withering and drying of plants as if they were suffering from drought. | Explained to the farmers about the occurrence of disease. Farmers are informed not to go for continuous cultivation of pigeon pea in the same field. Treat the seeds with Trichoderma viridi at 4g/kg of seed. Pull out the infected plants and burn them as this spread quickly - roughing or pruning diseased plants were advised to promote cost-effective treatment of SMD in pigeon pea. Neem oil spray (1 to 2 ml per Litre) or Profenophous (2ml/L) were recommended instead of expensive, strong chemicals. Farmers were advised not to go for spraying of chemicals inadvertently but adopt methods like pruning the infected plants, and only react with chemicals when the pest population reached the optimal threshold. The threshold was shown and discussed with farmers. Beyond the threshold, the hotline recommended organophosphorous chemicals which includes Carbofuran and Aldicarb for seed dressing, Carbofuran and Temik 10G for soil application, and Oxythioquinox, Kelthane, Dinocap, Monocrotophos, Tedion, and Metasystox as foliar sprays. |
| 12 | Farmers are not practising the use of green manure crops. | Farmers tend to leave the field fallow after Kharif crops. They are not aware of green manure crops for soil enrichment. | Created awareness about growing green manures that would enhance soil fertility and crop productivity. If pre-monsoon showers come (end of May), it is ideal for sprinkling seeds (1-2 kg) Sunhemp and allowing them to grow for 30 days. Incorporate this green manure into the soil by shallow tilling during the first week of July and go for pigeon pea/finger millet sowing after 7 to 8 days of incorporation. |
| 13 | Severe flower/bud drop is leading to decreased yield in pigeon pea. Profuse flowering but no pod formation. | Farmers are unaware of these physiological disorders. Farmers keep the same seeds for repeated cultivation. They also do not know plant growth regulators. | Explained the disorders and remedial measures. Spray PGR Planofix at 0.5ml per L or Agrigold (2ml/L) to control flower/bud dropping. They are also informed of changing seeds/varieties and following the seed treatment. |
| 14 | Nut dropping/Nut splitting in arecanut. | Lack of awareness about this physiological disorder. This disorder seen in young and healthy palms. Farmers may neglect this disorder. | Suggested farmers to collect all the infected nuts and other plant parts and destroy them. Farmers are also asked to improve drainage facilities and maintain proper management of the field. For nut splitting, they are recommended Borax (2g/L of water) during the early stages of the disorder. |
