# Supplementary material for: Harnessing digital technology to improve agricultural productivity?
Source: PLoS One. 2021 Jun 28;16(6):e0253377. doi: 10.1371/journal.pone.0253377 (PMC8238233; doi:10.1371/journal.pone.0253377)
Supplement: S6 Table — (DOCX) [file pone.0253377.s007.docx]

S6 Table. P-values for model with controls

| Outcome variables | Model P-value | Resample P-value | Romano-Wolf  p-value | Holm  p-value |
| --- | --- | --- | --- | --- |
|  | (1) | (2) | (3) | (4) |
| Log of crop yield  Log of profit  Log of revenue  Log of cost of production | 0.0191  0.0102  0.0132  0.5524 | 0.0010  0.0010  0.0020  0.5495 | 0.0050  0.0040  0.0050  0.5495 | 0.0020  0.0040  0.0060  0.5495 |

Notes: We follow D. Clarke, J. Romano, M. Wolf, The Romano-Wolf multiple hypothesis correction in Stata. IZA DP No. 12845 (2019) multiple hypothesis correction with four outcomes and one treatment.

**S1 Description. Description of the experimental intervention**

The experimental intervention has five stages. The treatment is simple and straightforward with the distribution of KCC helpline phone number to only the randomly selected treatment farmers.

*First stage*: Baseline survey (300 farmers/327 villages/1 districts). The study was carried out as part of the Dynamic Agricultural Tablet-based Extension Services (DATES) program. The research programme using randomised intervention aims to provide information on best agricultural practices, weather, credit, insurance and input and output prices with the objectives of enhancing crop yields, reduce the cost of cultivation and augment overall farm profitability. In the baseline survey, we collected detailed farming information for the previous crop cycle.

*Second stage*: A pre-experimental survey. We conduct face-to-face interviews among randomly selected farmers registered under the DATES program in Tumkur district to gauge the nature and extent of SMD infestation, quantification of expected loss of yield, source of seed purchased, source of any proposed solutions received, and awareness about the Kisan Call Centre (KCC) helpline extension phone number for advice. Note at the second stage we provided the KCC helpline extension phone number. It was clear from the survey that none of the farmers was aware of the KCC helpline phone number.

*Third stage*: Treatment (200 treatment farmers / 100 control farmers). We randomly allocated farmers to treatment and control groups. Only the treatment farmers were provided with the KCC helpline phone number and encouraged to call the number for support on any crop-related problems not just for SMD in pigeon pea.

*Fourth stage*: Follow-up survey. Follow-up surveys were conducted immediately after the harvest of the pigeon pea crop. We record information on several questions, such as, if the farmer called the KCC helpline. If not called, then the reasons for not calling. If called, what was the information received and if the advice received was followed. Did the adviser recommend any chemical sprays? Did the adviser provide the source and price of the chemical sprays? We also probe the usefulness and trustworthiness of the information.

*Final stage*: Endline survey. The endline survey gathered information on all crops grown by both treatment and control farmers. The information is related to crop yields, cultivating practices, detailed information on the cost of cultivation of each crop by farming operations, all sources of receipt of agricultural information, household assets owned, and farmer characteristics such as education, age, caste, and religion.
