# Supplementary material for: Harnessing digital technology to improve agricultural productivity?
Source: PLoS One. 2021 Jun 28;16(6):e0253377. doi: 10.1371/journal.pone.0253377 (PMC8238233; doi:10.1371/journal.pone.0253377)
Supplement: S1 Data — (ZIP) [file pone.0253377.s008.zip › SI data/ReadMe file for Helpline_dataset.docx]

**ReadMe** file for "Harnessing digital technology to improve agricultural productivity?"

**Data Files**

Two files containing data in excel format that can be called into STATA directly

1. HL_BasicBalance. xlsx
2. Helpline_Long_Regression Tables_Paper. xlsx

**Do File (STATA 13 format)**

Commands for replications.do file that does all balance tabulations, runs all regressions and generates figures reported in the main text of the paper and the tables reported in the SI Appendix. Each piece of code is clearly labelled and logically structured, not necessarily as presented in the paper. For instance, the baseline balance is performed first, and then the table and figures are shown in the paper's main text.

**Notes**

We note in the paper that each household in the experimental area produces more than one type of crop, which could be up to three different crops or the same crop but of different seed variety in a season. We exploit this cropping pattern within a farm household to improve the sample size, with total observations around 312 in the modelling. As the information required for crops varies, it is expected that the helpline would have a differential impact on different crops. Refer to file (a) to recompute balance check of the farm and farmer specific variable: Family size (number of members); Age in number of years; Education (number of years of education); Caste; Crop experience (number of years in crop farming); Land owned in acres; Total land cultivated in acres; Total land Irrigated in acres; Log(Total asset value)(i.e. household durables value); House owned; Car owned; Bike owned; Television owned; Radio owned; Bi-cycle owned; Telephone/ mobile owned; Number of visits of the Public Extension Advisor; Source of crop information: public/private; Household road distance to GP in km; Household road distance to State / National Highway in km; Household road distance to sub-district town in km. And, refer to file (b) to recompute the balance check of the crop-specific variable. Cropland cultivated in acres; Overall crop yield per acre; Overall crop yield per acre; Redgram yield/acre (overlap households); Ragi yield/ acre (overlap households); Horsegram yield/ acre (overlap households).
