# Supplementary material for: Harnessing digital technology to improve agricultural productivity?
Source: PLoS One. 2021 Jun 28;16(6):e0253377. doi: 10.1371/journal.pone.0253377 (PMC8238233; doi:10.1371/journal.pone.0253377)
Supplement: Attachment — Submitted filename: PONE-D-20-33119_review_comments.pdf [file pone.0253377.s009.pdf]

Review of PONE-D-20-33119 “Harnessing digital technology to improve agricultural productivity?”

Overall comments

This paper evaluates the impacts of SMS-based extension advisory services on yield outcomes on a sample of Indian farmers. This is an important question, given the fervor surrounding investments in digital agriculture in developing countries, and the immodest claims for such investments, which still often lack a solid empirical foundation. The experimental set-up has some nice features which address potential bias from self-selection and from treatment spillover. However, the nature of the information treatment and its linkage(s) with outcomes of interest is not clear enough for the analysis to be very compelling. If these aspects of the study can be clarified, I think the paper may make a nice contribution and merit publication in PLOS One.

Major issues

The main shortcoming of the paper is a lack of conceptual clarity about the nature of the information treatments and the outcomes of interest. Please be much clearer about the type of advice that is conjectured to have been most relevant for understanding these results, i.e. what advice did red gram farmers receive when calling in about an incidence of SMD? What are the other types of information provided and how are these different types of information linked to the outcomes of interest in this study? The current discussion around impacts of “better farming information” is far too vague. Somewhat relatedly, the discussion of adoption of “cost-effective” farming practices needs much more clarity for us to understand this. I suggest a section that clearly lays out the types of information provided, and its expected impacts on different outcomes of interest.

Given the importance of timing of advisory services, how is the timing of treatment controlled for in this analysis?

You should provide a fuller description of the intention-to-treat analysis -- i.e. that your analysis uses the initial treatment assignment (not the actual information treatment) as the basis for impact assessment. (At least, if I understand correctly what you did. It is not presented very clearly.) You should clearly show the estimating equation and the terms which constitute the intention-to-treat effect.

Your assertion that the information treatment is “variety specific” needs some unpacking. First, how would the advice for dealing with SMD differ by different red gram varieties? Second, given what we now know about widespread misidentification of varieties by farmers (e.g. Kosmowski et al. 2019, Jaleta et al. 2020), what implications might this have for your analysis?

It is not clear why the information treatment would affect cost of harvesting. Can you explain this? Also, what are interculture costs, and how would these be affected by in-season advisory services?

The review of related literature examining impacts of advisory services is very limited and needs to be expanded. Lots of experimental approaches to impact evaluation of digital advisory services have been coming out recently (e.g. Ayalew et al. 2020, Arouna et al. 2020 – see Norton and Alwang 2020 for a review of others).

What are the limitations of this study? What are the threats to identification in your analysis? What about external validity? These issues should be at least acknowledged.

Finally, if I understand correctly, in the absence of the SMD event, would there have been any detectable effects? Given the crop-specific results, I think not. That result deserves more discussion.

Detailed comments

General advice: use page numbers in submitted manuscripts to facilitate comments!

Abstract: “a high-stakes crop” – tell us what crop this is and what makes it a “high-stakes” crop.

Abstract: “Our findings reveal a [more] nuanced causal relationship than previously suggested, providing an explanation for the existence of mixed results [of prior impact evaluations].” See suggested insertions in brackets.

Abstract: is this a national study? If not, give a few more details of the study scope & sample size.

I think “redgram” should be rendered as “red gram”.

Can you give us an example of the kind of response advice that is given in response to early detection of SMD?

“Careful rouging of the infected plant…” Can you explain what this means? Do you mean roguing?

Many minor grammatical issues (e.g. dropped articles) suggest that professional copyediting may be desirable.

“The usage of the KCC helpline number was high, with 91% of the treated households calling the number to receive a range of information (online Appendix Figure S1)” – this is not clear, as the figure indicates treatment in terms of households, but uptake of advice is given as plot-crops. Is there just one plot-crop per sampled household?

For PLOS ONE’s readership, you should explain how the “intent-to-treat impact” is calculated.

Table 1: what is “Hotline impact”? do you mean use of the hotline, conditional on treatment?

You talk about “investing in new technology” (which I assume is referring to varieties and inputs) but we do not know if these technologies are in fact new for the farmers in this sample.

References

Arouna, Aminou and Michler, Jeffrey D. and Yergo, Wilfried and Saito, Kazuki, One Size Fits All? Experimental Evidence on the Digital Delivery of Personalized Extension Advice in Nigeria (2020). Available at SSRN: https://ssrn.com/abstract=3593878 or http://dx.doi.org/10.2139/ssrn.3593878

Ayalew, H., Jordan, C. and Newman, C., (2020). Site-Specific Agronomic Information and Technology Adoption: A Field Experiment from Ethiopia. Trinity College Dublin, Economics Department. https://EconPapers.repec.org/RePEc:tcd:tcduee:tep0620

Jaleta, M., Tesfaye, K., Kilian, A., Yirga, C., Habte, E., Beyene, H., ... & Erenstein, O. (2020). Misidentification by farmers of the crop varieties they grow: Lessons from DNA fingerprinting of wheat in Ethiopia. Plos one, 15(7), e0235484.

Kosmowski, F., Aragaw, A., Kilian, A., Ambel, A., Ilukor, J., Yigezu, B., & Stevenson, J. (2019). Varietal identification in household surveys: results from three household-based methods against the benchmark of DNA fingerprinting in southern Ethiopia. Experimental Agriculture, 55(3), 371-385.

Norton, G. W., & Alwang, J. (2020). Changes in Agricultural Extension and Implications for Farmer Adoption of New Practices. Applied Economic Perspectives and Policy, 42(1), 8-20.
