# Supplementary material for: Harnessing digital technology to improve agricultural productivity?
Source: PLoS One. 2021 Jun 28;16(6):e0253377. doi: 10.1371/journal.pone.0253377 (PMC8238233; doi:10.1371/journal.pone.0253377)
Supplement: Attachment — Submitted filename: Review of PONE-D-20-33119.pdf [file pone.0253377.s010.pdf]

Review of “Harnessing digital technology to improve agricultural productivity?” (PONE-D-20-33119)

1. Is the manuscript technically sound, and do the data support the conclusions?
    a. Yes but please see below for some suggestions on how to improve the paper.
    b. KCC hotline services section: How do things work when farmers call the KCC hotline? Do they speak live with a Level 1 operator and get answers to their specific questions? And how site-specific is the information provided? This information would greatly improve the reader’s understanding of the nature of the information that is provided by the KCC hotline.
    c. Nature of crop pest shock section: It is important to back up the reported yield losses and restorative measures described with adequate citations. There are no citations in this section at present.
    d. Minor point but given the international readership of the journal, I suggest using the terms pigeon pea and finger millet throughout (instead of redgram and ragi, respectively).
    e. Sample selection section:
        i. Why was Gubbi taluk selected and how generalizable are the results of the study to other areas (external validity)? What is the prevalence of mobile phone ownership in the study area?
        ii. What specific criteria were used to designate given areas as “predominantly redgram-growing, ragi-growing, and the rest”? How many of the treatment and control villages (and farmers) are in each of these strata?
    f. Data section: what exactly is meant by “crop-plots”? More information on the unbalanced panel is also needed in the main text. How many crop-plots are in the baseline survey? Of those, how many are also on the endline survey and how many new crop-plots on the endline survey? What is the breakdown by treatment vs. control and by crop? How many households control these crop-plots?
    g. Results section: If redgram was the only focal crop of the study to experience a significant pest shock during the study period, then I don’t follow the logic of this sentence: “The information from the hotline – recommendations based on Integrated Pest Management (IPM) methods – is expected to be equally useful for all crops and against most pests and diseases. Thus, the shock to redgram per se is unlikely to be the cause for differences in the crop yields.” A similar point is made in the Discussion that I disagree with: “However, information on IPM methods from hotlines is equally useful for all crops.” These points also seem to conflict many of the arguments made in the Discussion section in terms of why there are KCC impacts on redgram but not other crops. Had there been a pest shock to the other crops that could be addressed via IPM (or other methods on which technical advice could be provided over the phone), might we expect similar effects?
    h. Discussion section: I don’t find the closing paragraph useful or adequately supported with citations. I suggest dropping it and wrapping up the discussion with a concluding sentence or two at the end of the previous paragraph.

2. Has the statistical analysis been performed appropriately and rigorously?
    a. Yes, as far as I can tell. (I am not an RCTs expert.)
    b. The interpretation of the results throughout the paper as percentage point (pp) effects is incorrect. These are percentage effects, not PP effects, because the dependent variable is in logs. This needs to be corrected throughout the paper wherever results are discussed. See Wooldridge's Introductory Econometrics textbook (any version) for more info on how to interpret log-level models.
    c. Figure 1: it would be more accurate to label the lines treatment and control.
    d. It would be helpful to add error bars to Figure 2 and use 2-dimensional (instead of 3-dimensional) columns.

3. Have the authors made all data underlying the findings in their manuscript fully available?
    a. Yes, the data, replication code, and instructions are included in a zip file that can be linked to in the supplemental materials.

4. Is the manuscript presented in an intelligible fashion and written in standard English?
    a. Yes, the paper is generally well written but there are numerous grammatical errors. The paper (including the abstract) would benefit from careful copy editing.
