# Supplementary material for: Harnessing digital technology to improve agricultural productivity?
Source: PLoS One. 2021 Jun 28;16(6):e0253377. doi: 10.1371/journal.pone.0253377 (PMC8238233; doi:10.1371/journal.pone.0253377)
Supplement: Attachment — Submitted filename: Response to reviewer_2_19_Jan_2021.doc [file pone.0253377.s011.doc]

*Comments in italics*. Responses in regular font.

Responses to Reviewer #2

Thank you very much for your time and comments, which we believe have significantly improved the paper. Below we present the point-wise response showing how we addressed your comments in the revised paper.

- *KCC hotline services section: How do things work when farmers call the KCC hotline? Do they speak live with a Level 1 operator and get answers to their specific questions? And how site-specific is the information provided? This information would greatly improve the reader's understanding of the nature of the information that is provided by the KCC hotline.*
- Under the KCC hotline services, farmers call a typical, toll-free advisory phone number, to access expert advice from Level 1 operators (agricultural graduates) in 13 regional centres. The crop-specific extension information, provided from centres located across the country, is delivered in 21 local languages. Further, queries from Level 1 operators are supported by Level 2 experts, who are in different parts of the country at State Agriculture Universities, Indian Council for Agricultural Research Institutes, and State Department of Agriculture. Farmers speak live with the Level 1 operators and get answers to their questions immediately. If Level 1 operators are unable to provide answers, the calls are passed-on to Level 2 experts who also provide answers promptly. The solutions provided are plot- and crop-specific. We incorporate the above details in page 5 of the revised paper.
- *Nature of crop pest shock section: It is important to back up the reported yield losses and restorative measures described with adequate citations. There are no citations in this section at present.*
- In the revised paper (page 6), we have added four new references to back up the reported yield losses and restorative measures:

22. L. Kumar, T. Jones, D. Reddy, A novel mite-transmitted virus with a divided RNA genome closely associated with pigeonpea sterility mosaic disease. *Phytopathology* 93 (2), 71–81 (2003).

23. K. Ganapathy, M. Gowda, B. Ajay, S. Venkatesha, B. Gnanesh, S. Gomashe, P. Babu, G. Girish, P. Prasad, G. Veerakumar, J. Patil, Inheritance studies of sterility mosaic disease (SMD) resistance in vegetable type pigeon pea (Cajanus cajan (L.) Millsp.). *Australian Journal of Crop Science* 63, 1154–58 (2012).

24. L. Kumar, T. Jones, F. Waliyar, Biology, etiology and managment of pigeon pea sterility mosaic disease. Annual Review of Plant Pathology 3, 1-24 (2004).

25. T. Jones, L. Kumar, K. Saxena, N. Kulkarni, V. Muniyappa, F. Waliyar, Sterility Mosaic Disease - the 'Green Plague' of pigeon pea: advances in understanding the etiology, transmission and control of a major virus disease. *Plant Disease*, 88 (2), 436–445 (2004).

- *Minor point but given the international readership of the journal, I suggest using the terms pigeon pea and finger millet throughout (instead of redgram and ragi, respectively).*
- We agree with you. Given the journal's international readership, we changed the terms pigeon pea and finger millet instead of redgram and ragi.
- *Sample selection section: Why was Gubbi taluk selected and how generalizable are the results of the study to other areas (external validity)? What is the prevalence of mobile phone ownership in the study area?*
- While Tumkur was one of the districts preselected for implementing a large-scale agricultural program, we chose Gubbi for two reasons. (a) After completing the baseline survey under the agricultural program, we noticed the early signs of an SMD outbreak in Gubbi. (b) Access to the KCC hotline is available, but farmers are unaware of its services. The Gubbi sub-district is representative of pigeon pea growing areas across central and southern India. Any generalizations of the results beyond this region may be problematic. A study from the region suggests that only 87% of the households owned mobile phones. However, our baseline survey indicates 100% ownership among sample households owning one or more mobile phones (SI Table S4). We incorporate the above details in page 9 of the revised paper.
- *What specific criteria were used to designate given areas as "predominantly redgram-growing, ragi-growing, and the rest"? How many of the treatment and control villages (and farmers) are in each of these strata?*
- Two crops – pigeon pea and finger millet – proved significant in terms of area and main staples. To guarantee the desired heterogeneity in terms of crops, we stratified villages by their share of each crop area to total cultivated into three predominantly pigeon pea-growing, finger millet-growing, and the rest. Twenty-eight villages, primarily growing pigeon pea, were randomly allocated to treatment with twenty-five to control. We randomly assigned twenty villages with a significant share of finger millet to treatment and twenty-two villages to control. Rest of the twenty-six villages are split with eleven randomly allocated to treatment and the rest to control. The households in each stratum consist of 90 pigeon pea farmers, 107 finger millet farmers, and 38 growing other crops (see page 10 in the revised paper).
- *Data section: what exactly is meant by "crop-plots"? More information on the unbalanced panel is also needed in the main text. How many crop-plots are in the baseline survey? Of those, how many are also on the endline survey and how many new crop-plots on the endline survey? What is the breakdown by treatment vs. control and by crop? How many households control these crop-plots?*
- Here crop-plot refers to a parcel of land with a single crop demarcated by raised bunds.
- In the baseline survey, there are 193 crop-plots which also appears in the endline survey along with 42 additional crop-plots. Figure 1 in the revised paper shows that 160 treatment farmers cultivate 297 crop-plots, and 75 control farmers cultivate 131 crop-plots. The breakdown of crop-plots by type of crops are 148 pigeon pea, 208 finger millet, and 72 other crops. We include these details in the revised paper on page 11.
- *Results section: If redgram was the only focal crop of the study to experience a significant pest shock during the study period, then I don't follow the logic of this sentence: "The information from the hotline – recommendations based on Integrated Pest Management (IPM) methods – is expected to be equally useful for all crops and against most pests and diseases. Thus, the shock to redgram per se is unlikely to be the cause for differences in the crop yields." A similar point is made in the Discussion that I disagree with: "However, information on IPM methods from hotlines is equally useful for all crops." These points also seem to conflict many of the arguments made in the Discussion section in terms of why there are KCC impacts on redgram but not other crops. Had there been a pest shock to the other crops that could be addressed via IPM (or other methods on which technical advice could be provided over the phone), might we expect similar effects?*
- You make a very interesting point. Indeed, pigeon pea was the only focal crop to experience a significant pest shock. Non-pigeon pea crops had a regular agricultural season with many minor pest and disease issues such as mites, fungal infections, etc. The IPM methods over the hotline can be equally effective against these minor pests and diseases, but farmers chose to access hotline primarily to address SMD in pigeon pea (See SI Table S3). The low demand for hotline by farmers who cultivated only non-pigeon pea suggests that farmers value the extension information more when faced with the risk of crop loss. No detectable effect may be found in the absence of SMD. We find that farmers' access hotline only for the crop that faced a covariate shock and not for crops tackling minor pest problems. However, it may be likely that farmers were preoccupied with addressing a more significant issue in pigeon pea, thus ignoring the smaller issues in other crops, despite knowing the effectiveness of hotline information against pest and diseases in any crop. However, a discussion on what happens to the demand for hotline services in the presence of major shock to non-pigeon pea crops will require an entirely different experimental setup beyond our paper's scope.
- *Discussion section: I don't find the closing paragraph useful or adequately supported with citations. I suggest dropping it and wrapping up the discussion with a concluding sentence or two at the end of the previous paragraph.*
- As suggested, we dropped the closing paragraph and introduced a paragraph discussing the study's limitations, as suggested by the first reviewer. The closing paragraph is as follows: Our analysis's principal limitation is that we compare the hotline impact over two different crop cycles with the shock occurring only in the second crop cycle year. We use the difference-in-difference estimation strategy to address the concern, which compares the outcome before and after the SMD shock and between treatment and control groups. Another limitation of our study is the insufficient statistical power to examine the heterogeneous effect of hotline treatment by caste and land size holdings. More research on improving the extension system to be more inclusive will require a larger sample with improved research design. Please see pages 15 and 21 for details.
- *The interpretation of the results throughout the paper as percentage point (pp) effects is incorrect. These are percentage effects, not PP effects, because the dependent variable is in logs. This needs to be corrected throughout the paper wherever results are discussed. See Wooldridge's Introductory Econometrics textbook (any version) for more info on how to interpret log-level models.*
- Thank you for drawing our attention to this; it's an omission which we now correct throughout the document.
- *Figure 1: it would be more accurate to label the lines treatment and control.*
- Thanks for the suggestion. We now label the lines as treatment and control in Figure 1.
- I*t would be helpful to add error bars to Figure 2 and use 2-dimensional (instead of 3-dimensional) columns.*
- Thank you for the suggestion. The error bars are added, and the figure is changed to 2-dimension.
- *Yes, the paper is generally well written but there are numerous grammatical errors. The paper (including the abstract) would benefit from careful copy editing.*
- We thoroughly checked and corrected the paper for grammatical errors.
