# Supplementary material for: Harnessing digital technology to improve agricultural productivity?
Source: PLoS One. 2021 Jun 28;16(6):e0253377. doi: 10.1371/journal.pone.0253377 (PMC8238233; doi:10.1371/journal.pone.0253377)
Supplement: Attachment — Submitted filename: Response to Reviewer1_21_April_2021.doc [file pone.0253377.s012.doc]

*Comments in italics*. Responses in regular font.

Responses to Reviewer #1

Thank you very much for your time, and further comments, which we believe helped polish our paper's contribution. Below we present the point-wise response showing how we addressed your comments in the revised paper.

Comments:

Overall comments

- *This revision is improved over the previous version. The methods in particular are much better articulated and described. However, there are still some issues to be addressed.*
- *In particular, you still need to better situate this paper within the literature. You say on page 3 "Our study contributes to the evolving experimental literature on the impact of digital advisory services in agriculture (12-14)." But you do not follow up by telling us what this contribution is, relative to the existing literature. Is it simply the same research question and methods applied to a new empirical context (i.e. a different intervention in a different geographical setting)? Or something different? In general, you should try to help readers know how to cite your paper. Is it simply another example of evidence for a positive impact of advisory services? Or something a bit more nuanced?*
- P3-5: In the Introduction, we summarized our main results and then listed two contributions of our paper. The following are the additions to the revised paper:
- Our main result shows that access to better farming information on damage control increases crop yield by 31% with the adoption of cost-effective and improved farming practices. Additionally, we have two key findings from the disaggregate analysis. First, access to the hotline increases pigeon pea yield by 87% for the treatment farmers relative to the control group with no access to the hotline. Second, though the overall costs of cultivation increased by 39% from intervention, profits for the treatment farmers cultivating pigeon pea are higher by 70%. The observed impact is understandable given the nature of pest shock in the pigeon pea crop and the timing and significance of the expert advice received via the hotline in reducing the losses for the treatment group.
- Our study contributes to the evolving literature on the impact of digital advisory services in agriculture in two ways. First, we present new evidence on improving the delivery of agricultural extension services in developing countries (12-14). In existing experimental studies, the provision of agricultural advisory services is still heavily supply-driven (13). However, observational studies have shown that the demand for extension services may not be high (15, 16). For instance, a recent study on Malawi suggests that access to agricultural advice does not necessarily lead to greater crop productivity (17). Those quality extension services, measured in terms of the farmer's perceived usefulness and relevance of the advice, are a significant predictor of agricultural productivity at the plot level. Thus, with low demand, the supply-side intervention with even good quality extension services is unlikely to influence the outcome, though also dependent on the type of crop and nature of crop shock. Our intervention provides access to reliable extension information and differentiates the demand by crop shock to gauge the impact on agricultural productivity. Our paper offers the first credible evidence showing that the need for extension services is high only when faced with significant crop losses.
- Second, we provide evidence that failure to account for heterogeneity in crop-specific shocks may be a limiting factor in adopting improved technology. This result suggests that customized shock-specific recommendations could improve crop productivity relative to existing generic recommendations. There is no empirical study to the best of our knowledge that assesses whether information accounting for heterogeneity in crop-specific shocks results in higher yields and profits. While extension services are already prevalent in many countries, improving their access and timely delivery with customized information to overcome idiosyncratic crop shocks can accelerate agricultural productivity.
- *Relatedly, in the discussion or conclusions, you may compare/contrast your main analytical conclusions with those of other empirical papers in the literature.*

- P23-24: While listing the contributions in the Introduction as stated above, we also try to compare/contrast our main results to the existing studies. In addition, we add a "Conclusion" section as suggested.

**Conclusion**

Traditionally agricultural extension is delivered by extension workers with often no training in science-based agricultural advice, limited budget to manoeuvre farm visits and poor accountability. With digital agriculture, governments can reorient their policies and budgets to strengthen transmissions of information electronically. The broader coverage of mobile phone networks has enhanced the potential to address the information asymmetries within poor communities at no additional costs. Our paper explores whether we can improve the adoption of best agricultural practices by making information supply and acquisition less costly by harnessing the existing communication infrastructure.

Several past studies have examined the impact of access to agricultural information, reporting mixed results and considerable context-dependence. Our paper builds on these studies by going beyond access, suggesting a need to pay close attention to the heterogeneity of crop shocks and information delivery timing. Thus, for crops that faced adverse shocks, the demand for information is likely high, so the information delivery could positively impact productivity. In contrast, for crops with moderate shock, provisioning of information may not affect output due to the low demand for extension services. Our results reveal a more nuanced causal relationship than previously suggested, explaining the existence of mixed results.

- *Furthermore, the main analytical conclusions are not always clear. For example, in the discussion on page 21, you state "This study documents that ICT tools do not unequivocally increase productivity, so merely scaling up the ICT infrastructure for improving access to extension information may be insufficient to enhance agricultural productivity." But you do not tell us what is required in addition to (or in place of) scaling up. As a result, it is hard to know what to make of this conclusion.*
- P22: We clarified as 'Locally customized information delivered at the appropriate time during the farming season or sending messages targeting specific pest outbreaks can augment the demand for extension services.'
- *Similarly, on page 21, you state: "While extension services are already prevalent in many countries, improving their access and timely delivery with new ICT tools can accelerate agricultural development." Are you simply advocating for a move from analog to digital extension methods? Or something more specific? Basically, as currently written, it is not clear what conclusions the reader should come away with.*

- P23: To clarify, we added the following statement in the revised paper: 'For instance, sharing photographs electronically (e.g., posters) using Android-based devices to identify and confirm specific agricultural problems would increase the effectiveness of hotline services.'

- *Finally, I could not see how or where to access the data and Stata code, although the submitted materials indicate that these are available as supplementary materials.*
- We deposited all the dataset and the STATA code required to replicate the results on the PLOS ONE website as part of the submission of the manuscript. It is available on their website, but we assume that it will be linked to the Supplementary materials once the paper is published.

*Detailed comments*

- *The paper still needs proofing! Some examples from the abstract ("we randomly distributed hotline number" missing an article; "results show that eliminating informational inefficiencies increase..." where the last word should be "increases").*
- Thanks for the examples. The other corrections completed in the revised paper are as follows:
- P2: ".. to control spillover effects and contaminate the control group."
- P3: "More than just access to information, the timing of the delivery of expert advice may be crucial in farming.". Here "may be" is moved from the beginning of the sentence to towards the end.
- P8: "However, on average, farmers have 30 years…".
- P9: "..the hotline…". Here "the" was added. Missing an article was a common mistake throughout the paper that is now corrected.

Here are few examples. There are too many corrections made in the revised paper for it to be all listed here.

- *P3: "Our results have considerable policy relevance…" If you claim to have policy relevance, you must say what this is very explicitly. What specific new insight or insights for policy have been gained from this study?*
- P23-24: Instead of saying "policy relevance," which we deleted from the revised paper, we have now explicitly stated the relevance. The following text has been added: "Traditionally agricultural extension is delivered by extension workers with often no training in science-based agricultural advice, limited budget to manoeuvre farm visits and poor accountability. With digital agriculture, governments can reorient their policies and budgets to strengthen transmissions of information electronically."
- *P3: "The broader coverage of mobile phone networks can address the information asymmetries within poor communities at no additional costs." What does this mean? Are you suggesting that simply adding more cell phone towers will reduce information asymmetries facing farmers? That does not make sense.*
- P24: We are not suggesting but pointing out the following in the revised paper: "The broader coverage of mobile phone networks **has enhanced** the potential to address the information asymmetries within poor communities at no additional costs." Please note that in the revised paper, we have moved this sentence to page 24.
- *P21: "Considerable rethinking in innovative ways is critical to enhancing the demand for extension services." What does this mean?*
- P22-23: We clarified by elaborating on how the demand for extension services can be enhanced in the revised paper. We incorporated the following text in the revised paper: "To enhance demand, reminders, and information nudges via farmer schools or workshops with demonstration plots can give the farmers confidence in digital agricultural advice.".
