# Supplementary material for: Harnessing digital technology to improve agricultural productivity?
Source: PLoS One. 2021 Jun 28;16(6):e0253377. doi: 10.1371/journal.pone.0253377 (PMC8238233; doi:10.1371/journal.pone.0253377)
Supplement: Attachment — Submitted filename: Response to Reviewer1_31_May_2021.doc [file pone.0253377.s014.doc]

Comments in regular font. Responses in red.

Responses to Reviewer #1

Thank you very much for your time and further comments, which we believe helped polish our paper's contribution. Below we present the point-wise response showing how we addressed your comments in the revised paper.

Comments:

I appreciate that the author tried to respond in good faith to the previous requests. However, it seems that the author rushed these revisions, as they often entail incomplete sentences (e.g., p 23) or very vague assertions. The manuscript text (including the revised sections) remains imprecise in many places, which makes for frustrating reading. Examples from the new paragraphs which were added in response to the request to clarify the contribution (p 4-5):

"Those quality extension services, measured in terms of the farmer's perceived usefulness and relevance of the advice, are a significant predictor of agricultural productivity at the plot level." What are "those quality extension services"? Not at all clear what this means. (I think you want to say something like: Farmer perceptions of the usefulness and relevance of received advice are significant positive correlates of agricultural productivity outcomes.)

- In the revised paper, we further clarified accepting changes suggested by you. The changes are as follows: "Farmer perceptions of the usefulness and relevance of received advice are significant positive correlates of agricultural productivity outcomes.".

"Thus, with low demand, the supply-side intervention with even good quality extension services is unlikely to influence the outcome, though also dependent on the type of crop and nature of crop shock." What outcome? Adoption of advice? Yield? Something else?

- Here, the outcome is the crop yield. Thus, we changed the sentence to as follows: "Thus, with low demand, the supply-side intervention with even good quality extension services is unlikely to influence crop yield, though also dependent on the type of crop and nature of crop shock."

"Our intervention provides access to reliable extension information and differentiates the demand by crop shock to gauge the impact on agricultural productivity." There are so many things going on in this sentence that its hard to parse. What does "reliable" mean in this context? What does it mean to differentiate demand by crop shock?

- Indeed, we agree; thus, this sentence is replaced with the following: "Our intervention provides advisory recommendations, delivered via a hotline, which is tailored to time- and crop-specific shocks to gauge the impact on agricultural productivity."

"Our paper offers the first credible evidence showing that the need for extension services is high only when faced with significant crop losses." I think you are confusing "need" with "demand". Furthermore, the claim itself is so broad that it's hard to take seriously. All farmers face crop losses. A much clearer and more defensible claim would be to say something like: Our results show that the demand for extension services increases when extension messaging directly relates to specific shocks, and is provided during the window of time in which responses to those shocks are feasible.

- We accepted your suggestion and replaced it with, "Our results show that the demand for extension services increases when extension messaging relates directly to specific shocks and is provided during the window of time in which responses to those shocks are feasible."

I think your whole point should be much simpler: your analysis indicates that advisory recommendations, delivered via a hotline, which are tailored to time- and crop-specific shocks are associated with greater impacts than generic recommendations. This is important and sufficient. It just needs to be stated clearly. You might also note that your results are consistent in spirit with other evaluations of tailored recommendations already cited (which indicate that tailoring recommendations to geographic and/or farmer-specific needs are associated with greater impacts than generic recommendations). However, you should more clearly acknowledge that your study focuses on a hotline service, and differs from many other evaluations of ICT-enabled advisory services which focus on digital advisory services provided via extension agents in the field.

- Thank you for the suggestion. We rewrite the entire section on page 5. The following are the changes: "Second, we provide evidence that failure to account for heterogeneity in crop-specific shocks may be a limiting factor in adopting improved technology. There is no empirical study to the best of our knowledge that assesses whether information accounting for heterogeneity in crop-specific shocks result in higher yields and profits. Our result suggests that customized shock-specific recommendations could improve crop productivity relative to existing generic recommendations (18, 19). Our result is consistent with the recent evaluation suggesting that personalized, localized advice is far more effective than blanket recommendations (13,14). Our study differs from these studies that focus on digital advisory services provided via extension agents in the field, while our evaluation utilizes hotline phone services."

Detailed comments:

P 23: This new sentence is incomplete: "Locally customized information delivered at the appropriate time during the farming season or sending messages targeting specific pest outbreaks."

- We rewrite this sentence as follows in the revised paper: "For augmenting the demand for extension services, hotlines can deliver locally customized information at the appropriate time during the farming season or by sending messages targeting specific pest outbreaks."

P 25: "In contrast, for crops with idiosyncratic shocks, the information provided may not affect output due to the low demand for extension services." What do you mean by idiosyncratic shocks? Do you mean shocks that are so localized that it is not possible to program advice for? Or are you basically referring simply to normal production conditions?

- Here, we mean localized production shocks. Thus, we replaced "idiosyncratic" with "isolated, localized production".

P 15: "Our results reveal a more nuanced causal relationship than previously suggested, explaining the existence of mixed results." I do not think this is true. What your study does is focus on a hotline which provides advice on how to respond to disease, which is categorically different from advisory services which focus on nutrient management or other routine aspects of crop husbandry. To say that your study is more nuanced than other studies in the literature is to mischaracterize the salient differences between your study and prior studies.

- We agree with your suggestion, thus, removed this statement from the text.

The abstract needs work to be coherent and understandable. For example:

"Recent evidence suggests both positive and insignificant ways in which SMS-based agricultural information could affect farming outcomes." → Do you mean both positive and negative, or both significant and insignificant?

- We mean significant and insignificant ways. Thus, the text is changed in the abstract (page 1) and introduction (page 2).

"Using methods from experimental economics, we randomly distributed the hotline number to generate exogenous variation in the access to farming information." → You need to describe "the hotline number" before this sentence for us to understand it.

- We introduced the following sentence that describes the hotline services. "Hotline services provide rapid, unambiguous information by agricultural experts over the phone, which are tailored to time- and crop-specific shocks."

"Our findings reveal a more nuanced causal relationship than previously suggested, explaining the existence of mixed results." → I don't think you can claim to explain mixed results of impact evaluations of digital extension tools in a literature that spans many different types of tools and contexts. This may be part of an explanation, but you cannot claim to categorically explain the mixed empirical results in the literature without further qualifications.

- We agree with your suggestion thus replaced the sentence with the following: "Our findings reveal that advisory recommendations customized to time- and crop-specific shocks are associated with greater impact on agricultural productivity."
